# Effectiveness of a Pharmacist-Led Web-Based Medication Adherence Tool With Patient-Centered Communication: Results of a Clustered Randomized Controlled Trial

**DOI:** 10.2196/16141

**Published:** 2022-04-07

**Authors:** Jan van Lieshout, Joyca Lacroix, Aart van Halteren, Martina Teichert

**Affiliations:** 1 Scientific Center for Quality of Healthcare (IQ healthcare) Radboud Institute for Health Sciences Radboud University Medical Center Nijmegen Netherlands; 2 Department of Brain, Behavior & Cognition Philips Research Eindhoven Netherlands; 3 Department of Chronic Disease Management Philips Research Eindhoven Netherlands; 4 Department of Clinical Pharmacy and Toxicology Leiden University Medical Centre Leiden Netherlands

**Keywords:** medication adherence, improvement, intervention, web-based, tailored intervention, patient centered, barriers, primary care, cardiovascular diseases, diabetes

## Abstract

**Background:**

Growing numbers of people use medication for chronic conditions; nonadherence is common, leading to poor disease control. A web-based tool to identify an increased risk for nonadherence with related potential individual barriers might facilitate tailored interventions and improve adherence.

**Objective:**

This study aims to assess the effectiveness of a newly developed tool aimed at improving medication adherence.

**Methods:**

We performed a cluster randomized controlled trial in patients initiating cardiovascular or oral blood glucose–lowering medication. Participants were recruited from community pharmacies. They completed an online questionnaire comprising assessments of their risk for medication nonadherence and subsequently of barriers to adherence. In pharmacies belonging to the intervention group, individual barriers displayed in a graphical profile on a tablet were discussed by pharmacists and patients with high nonadherence risk in face-to-face meetings and shared with their general practitioners and practice nurses. Tailored interventions were initiated by pharmacists. Barriers of control patients were not presented nor discussed and these patients received usual care. The primary outcome was the effectiveness of the intervention on medication adherence at 8 months’ follow-up between patients with an increased nonadherence risk from the intervention and control groups, calculated from dispensing data.

**Results:**

Data from 492 participants in 15 community pharmacies were available for analyses (intervention 253, 7 pharmacies; control 239, 8 pharmacies). The intervention had no effect on medication adherence (B=–0.01; 95% CI –0.59 to 0.57; *P*=.96), nor in the post hoc per-protocol analysis (B=0.19; 95% CI –0.50 to 0.89; *P*=.58).

**Conclusions:**

This study showed no effectiveness of a risk stratification and tailored intervention addressing personal barriers for medication adherence. Various potential explanations for lack of effectiveness were identified. These explanations relate, for instance, to high medication adherence in the control group, study power, and fidelity. Process evaluation should elicit possible improvements and inform the redesign of intervention and implementation.

**Trial Registration:**

The Netherlands National Trial Register NTR5186; https://tinyurl.com/5d8w99hk

## Introduction

Adherence to chronic medication is problematic, leading to poor disease control with a burden on patients’ quality of life and health care systems [1]. Studies show that 17%-80% of patients with a chronic condition were not adherent, especially in asymptomatic conditions [2-6]. Various causes can hamper adherence and additionally, adherence varies between types of diseases and within patients over time [4,7,8]. Vrijens et al [9] made a taxonomy of nonadherence based on phases in the process of medication use: initiation, implementation, and persistence.

The multifaceted nature of the adherence problem illustrates that improving adherence needs interventions that are tailored to the individual patient [7,10]. Accordingly, recent high-quality randomized controlled trials in a systematic review on interventions for enhancing medication adherence tailored their interventions. These methods of improving medication adherence for chronic health problems were mostly complex and lacked effectivity [11]. In an overview of systematic reviews, Ryan et al [12] found self-monitoring of medicines and self-management programs to be generally effective. Simplified dosage regimens and pharmacists involvement in medication reviews and pharmaceutical care services on adherence involving patient education on good medication use were considered promising.

Systematic reviews showed that the pharmacist can play an important role in the prevention of cardiovascular diseases, mainly through patient education and counseling, drug safety management, medication reviews, monitoring and reconciliation, detection and control of risk factors, and clinical outcomes [13,14]. Community pharmacies are a natural location for patient recruitment at first dispensing to target patients with an increased risk for nonadherence and to perform a tailored intervention at the second dispensing moment.

In recent reviews nonadherence was reported to be high for cardiovascular and oral blood glucose–lowering medication [15-18]. In several studies, the risk for nonadherence was shown to be the highest in the first year after the start of chronic medication [19,20]. Consequently, interventions to warrant adherence are potentially most effective at the initiation of a chronic medication treatment.

At present, there is no tool that combines selection of those patients who are at risk for nonadherence and assessment of their individual barriers for good medication use in combination with offering tailored interventions by care professionals to overcome individual barriers. We have now developed a user-friendly medication adherence tool comprising a nonadherence risk and barrier assessment in an online patient questionnaire, pharmacists’ equipment to perform a tailored intervention based on a graphic barrier profile, and the intervention itself.

The primary research question reported in this paper was: What is the effectiveness of using the medication adherence tool on medication adherence of patients starting with cardiovascular or oral blood glucose–lowering medication identified as being at high risk for nonadherence at 8 months’ follow-up compared with usual care? Medication adherence was measured by pharmacy dispensing data. Furthermore, we assessed predictive values of the medication nonadherence risk assessment and the barrier questionnaire. Parallel to the effectiveness evaluation, we performed a process evaluation. In this publication we only report on the effectiveness evaluation.

## Methods

### Study Design

This was a cluster randomized trial with an intervention group of pharmacies (using the medication adherence tool) and a control group of pharmacies (providing usual care). The study design is explained in detail in the study protocol [21]. The patient inclusion period was from 2015 to 2017.

### Study Setting

In the Netherlands, the vast majority of the patient groups included (diabetes, cardiovascular risk management) is treated in primary care. General practices provide cardiovascular risk management and diabetes care, generally supported by care groups.

In the Netherlands, at the start of chronic medication patients with a first dispensing usually receive medication for 2 weeks. A second dispensing after 2 weeks is intended to assess first patient experiences with the drug. Only when the patient is willing to use the drug chronically, a follow-up dispensing for mostly 90 days is supplied. Consequently, patients starting with the study medication (intervention group) were expected to have a second dispensing after 2 weeks. The follow-up dispensing of chronic medication is expected to take place every 3 months.

### The Medication Adherence Tool

#### Overview

The medication adherence tool developed was based on the emotional, cognitive, and practical components in nonadherence. It comprised 3 elements: a patient questionnaire, pharmacy equipment, and the tailored intervention. All patients filled the questionnaires; however, the intervention and control groups differed in patient information from the questionnaire available to the pharmacist at the second dispensing, additional pharmacy training, and recommendations to address potential risk and barriers by a tailored intervention.

#### Patient Questionnaire

The online questionnaire consisted of 2 parts. First, the Probabilistic Medication Adherence Scale (ProMAS) measuring the nonadherence risk. The ProMAS is an 18-item validated questionnaire to assess nonadherence behavior in general [22]. One question from the original questionnaire was excluded because the validation study results showed that it had a substantially lower model fit than the other questions [22]. Two other questions needed to be excluded for those patients who did not already use medication chronically at that point of time. To know whether that was the case for the specific patient, we preceded the ProMAS with a question on whether the patient was on medication already. If answered with “no,” the 15-item questionnaire was presented excluding the questions about longer medication use; otherwise the 17-item questionnaire was presented. Patients were invited to participate in the study at the first dispense. Participating patients received the questionnaire shortly before the second dispense. If it appeared that they had not answered the questionnaire at the second dispensing moment, they were offered to answer the questionnaire in the pharmacy. Consequently, patient’s answers evaluated their experiences in medication adherence for at least 10 days.

The items consist of statements with yes/no answer categories. Examples of statements are “It has happened at least once that I forgot to take (one of) my medicines”; and “When I am away from home, I occasionally do not take (one of) my medicines.” The items skipped related to making changes in the medication use and being late for refills, as these questions consider a longer experience in chronic medication use. One original question considering longer medication use (“In the past month, I have forgotten to take my medication at least once”) was adapted to “Since the first dispensing, I have forgotten to take my medication at least once.”

Second, the barrier questionnaire measured to what extent the following emotional, cognitive, and practical barriers for nonadherence were present: feelings with regard to medication (emotional), fear of side effects (emotional), concerns about medication usage (emotional and cognitive), necessity beliefs (cognitive), attitude with regard to medication (cognitive), self-efficacy (cognitive and practical), inconvenience (practical), and applying the medication scheme (practical). The barrier questionnaire was developed and validated in an earlier study (data not shown). First, a list of 25 adherence determinants was composed, based on existing evidence in the literature about their relationship with medication nonadherence [8,23-27]. Second, an extensive questionnaire was composed by assessing this list of 25 determinants making use of existing (shorter versions of) instruments for the constructs for which they existed and self-composed items when instruments did not exist. Third, the questionnaire was administered to 1247 patients taking medication for their chronic condition in the Netherlands and United Kingdom. Furthermore, their medication adherence was assessed through pharmacy refills. Finally, based on predictive modeling, the determinants that were significantly predictive of medication nonadherence were selected and the less predictive ones were excluded. This resulted in a short and manageable questionnaire that included 24 items that screen for a set of 8 determinants (named barriers) that have been shown to have a significant impact on medication nonadherence. The questionnaire entails a screening for the potential presence of the barriers in the patient rather than a validated assessment. The result is verified in the conversation between the patient and the care provider. Examples of items and answering categories are as follows:

- In the domain self-efficacy: “If I do my best, I will succeed in taking my medicines according to my doctor’s prescriptions.” Answering categories: Strongly disagree/ Disagree/ Uncertain/ Agree/ Strongly agree.
- In the domain attitude: “How positive or negative are you about your prescribed medication?” Possible answers: Negative/ Somewhat negative/ Neutral/ Somewhat positive/ Positive.
- In the domain feelings: “I feel that I would rather stay away from my medication.” Answering categories: Strongly disagree/ Disagree/ Uncertain/ Agree/ Strongly agree (scored reversed).

#### Barrier Profile

**Figure 1:**
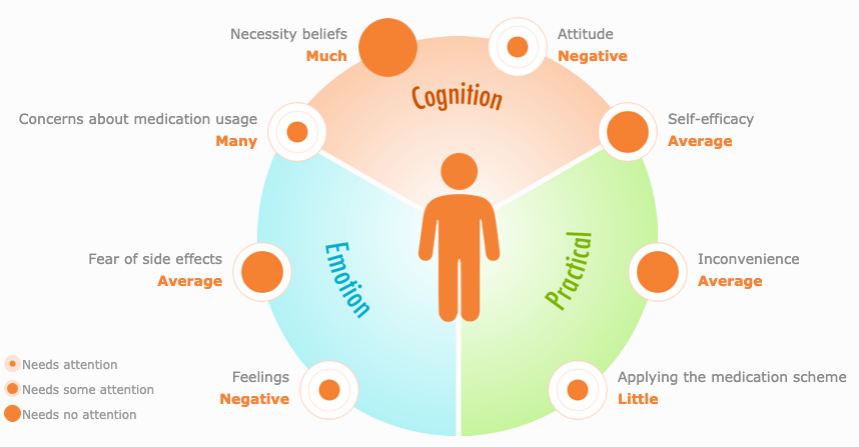
An example of a barrier profile.

The answers to the barrier questionnaire were translated into a visual barrier profile that presents each domain as a circle: a small circle corresponding to a barrier “asking for much attention”; a larger circle corresponding to “asking for some attention”; and the largest circle corresponding to “no barrier present” (Figure 1). The visual representation of the profile deliberately showed the largest circle when no barrier was found, to emphasize a patient’s strengths (full circle) and represent barriers as opportunities for growth (from small to larger circles).

The profile shows 8 potential adherence barriers: 2 emotional barriers (*feelings with regard to medication* and *fear of side effects*), 1 emotional/cognitive barrier (*concerns about medication usage*), 2 cognitive barriers (*necessity beliefs* and *attitude with regard to medication*), 1 cognitive/practical barrier (*self-efficacy*), and 2 practical barriers (*inconvenience* and *applying the medication scheme*).

#### Pharmacy Equipment

Intervention pharmacists and their pharmacy assistants received a 3-hour training comprising an introduction to patient-centered motivational communication, a demonstration of a second dispense discussion with a barriers profile, and skills practice using role play. The training was provided by a psychologist with expertise in patient-centered communication technique. The psychologist instructed the intervention pharmacists and their pharmacy assistants on discussing the profile with the patients to tailor the intervention to overcome personally relevant cognitive, emotional, or practical barriers.

Intervention pharmacies received a manual containing instructions for discussing and overcoming the various potential barriers as reference material at their disposal when they would need it. This manual was developed based on principles of patient-centered communication and with experts’ input. The manual held recommendations on how to address each of the possible barriers. In the developmental phase a stakeholder group was involved. This group comprised pharmacists, general practitioners (GPs), a communication expert, technicians, and researchers.

Each pharmacy was provided with a tablet that had an app (BOMM) installed, which was specially developed for registration of participating patients.

It also offered the opportunity to the patient to fill out the questionnaire at the second dispensing as a second chance to those who wanted to participate but failed to do so before. In the intervention pharmacies, the pharmacist used the tablet to review the graphical barrier profile during the second dispensing moment, and to make any notes regarding the conversation and applied intervention. Control pharmacies did not receive the information from the questionnaires, and their consultation during the second dispense was performed as usual.

#### Tailored Intervention

At the second dispensing moment, a tailored intervention was initiated for patients in the intervention group with an increased risk for nonadherence. The intervention started with a presentation and discussion of the barriers profile. In a personal face-to-face consultation, the pharmacist discussed the relevant barriers in the profile trying to take away or diminish these barriers using a patient-centered communication technique. The pharmacist could use the manual as a reference source to rely on for addressing the relevant barriers (the manual contained specific instructions for addressing each barrier). To address, for instance, the cognitive barrier of necessity beliefs, the intervention, for example, focused on emphasizing the necessity of the medication or stimulating the patient to start self-monitoring of blood pressure or blood glucose levels to make the effect of the medication more visible to him/her and thereby improve the belief that it is necessary to take the medication. Self-monitoring devices were made available by the research group for patients to use during the study. To overcome the practical barrier of a lack of applying the medication scheme, the pharmacist could give additional explanation about the medication scheme or a pill organizer or multidose drug dispensing systems; in some cases, a simplification of the dosage scheme could also be offered. The pharmacist could plan a follow-up consultation.

The pharmacist registered the intervention type in the automated information system, which is shared with the pharmacy team and the GP. This enabled the health care professionals in the general practice to take notice of the intervention and to pay attention to the adherence in line with the pharmacists’ intervention.

The feasibility of implementing the use of the adherence tool in the daily pharmacy workflow was piloted in 2 intervention pharmacies. Based on the experiences during the pilot period, workflow adaptations to increase convenience and efficiency were made. Study information for patients and pharmacists, comprising clarification for the patients and flowcharts for the pharmacies, was adapted after the pilot period based on feedback from the pilot pharmacies.

### Implementation of the Medication Adherence Tool

Members of the research team visited all participating pharmacists to explain the study in detail and the use of the app on the tablet and to provide them with an easy-to-understand explanation of the workflow concept. They subsequently also discussed how the workflow concept could be optimally tailored to existing routine work procedures in their pharmacy. One of the project group members from the care group was available during working hours to answer questions arising from the study. Finally, pharmacists had the opportunity to contact one of the pilot pharmacists for questions and advice about study procedures.

Furthermore, intervention pharmacies received a follow-up group session from the trainer on the patient-centered communication technique to provide support and exchange experiences on discussing the barriers profiles.

### Recruitment of Pharmacies, General Practices, and Patients

#### Drug Classes and ATC Codes

The trial was carried out with community pharmacists, GPs, and their patients with a first prescription of cardiovascular or oral blood glucose–lowering medication with Anatomic Therapeutical Chemical (ATC) codes A10B, B01AC, C01A, C01D, C03, C07, C08, C09, or C10 within the study period (see Multimedia Appendix 1 for drug classes and ATC codes and [28]). A first prescription was defined as no drug dispensing from that drug class to the patient in the preceding year. In the Netherlands, patients are listed with a GP. Furthermore, patients generally receive their medication from 1 community pharmacy.

The primary care collaborative DOH (De Ondernemende Huisarts or The Innovative General Practitioner) recruited the pharmacies and general practices for this study. DOH is a general practice collaborative in the South of the Netherlands that developed and implemented structured care for several prevalent chronic conditions including cardiovascular diseases and diabetes. The DOH works in close collaboration with the regional pharmacist organization Stichting Categorale Zorg voor apothekers in Zuidoost Brabant (CaZo). During the trial period patient inclusion failed compared with the expected numbers. For that reason we allowed pharmacies to also include patients listed in general practices not part of the DOH collaborative.

#### Pharmacies

DOH closely cooperates with 25 pharmacies and invited all for the study. In a pilot phase, 2 pharmacies tested the use of the medication adherence tool in their daily practice; for that reason they participated in the intervention group. The other participating pharmacies were randomly assigned to the intervention or control group by drawing lots (performed by an independent research assistant), and were informed about their assignment. To ensure that small and large pharmacies were evenly spread over the intervention and control groups, pharmacies were stratified by “size,” dichotomized as “large” or “small” (ie, the number of registered patients from DOH GPs).

#### General Practices

DOH general practices received written information about the study before the start. They were encouraged to contact a member of the research team for posing their questions in case things were unclear.

Furthermore, they were offered waiting room materials including information on the study for narrowcasting, to raise awareness about the study, and thus enhance patient inclusion. During the study period, GPs were informed about the study progress in terms of number of participants recruited. The barrier profile and the pharmacists’ notes on the intervention were available to the GPs and practice nurses in the electronic chain system, so they could build on this information related to these patients in the intervention group to increase medication adherence in their follow-up contacts with the patient.

#### Patients

Patients (>18 years) from a DOH GP with a first dispensing of cardiovascular or oral blood glucose–lowering medication (ATC codes mentioned above), without cognitive impairments, and able to read and speak Dutch were eligible for inclusion. The pharmacies’ automatized computer system alerts the pharmacist at first dispensing of any drug at the ATC level 5 (eg, simvastatin). However, the inclusion criterion for this study was more strict in defining a first prescription within a drug class (ATC level 3, eg, statins). Consequently, upon receiving an alert for a first dispensing, the pharmacist had to check whether the prescription was related to the medication studied and whether this dispensing concerned the first drug that the patient received within the drug class during the last year. Pharmacists informed eligible patients about the study, provided them with an information package that also contained the informed consent form, and invited them to participate. Those interested in participating were registered. We included only those patients who returned a signed informed consent form. One week after the first dispensing, these patients received a link to the online questionnaire by email or a paper-based questionnaire if preferred.

For all patients starting with cardiovascular or oral blood glucose–lowering medication with a first prescription from a DOH GP, the GP starts a treatment plan in the electronic information system (referred to as chain information system) that GPs and pharmacies use in their current daily practice to report about the condition and laboratory results of their patients and this is accessible to the different caregivers in the chain who are involved in the care of the patient (eg, GP, pharmacist, nurse practitioner). For patients in intervention pharmacies with a high nonadherence risk, the pharmacists could access the graphical barrier profile (see Figure 1 for an example) in the chain system and in the app on the tablet by entering the credentials of the patient.

### Ethical Approval and Informed Consent

The study was reviewed and approved by the local Medical Research Ethics Committee, the Commissie Mensgebonden Onderzoek (CMO) region Arnhem-Nijmegen (registration number 2015-1604).

During their pharmacy visit for a first dispense, eligible patients were invited to participate and received a package with study information and an informed consent form. Additionally, we asked informed consent from participating pharmacies and GPs to share data regarding the condition and the medication use of participating patients.

### Measures

#### Nonadherence Risk

The revised ProMAS questionnaire consisted of 15 or 17 questions depending on the use of chronic medication prior to the study. Each question on the ProMAS has 2 answer categories, leading to a maximum sum score of 15 or 17. In the 18-item ProMAS the cut-off value for being at risk for nonadherence was 14. As we skipped 1 question, we pragmatically lowered the cut-off value to 13: patients with a score of ≤13 were classified as having an increased risk for nonadherence, whereas those with a score above 13 were not. We used the same cut-off for patients who had to answer only 15 questions to minimize the probability of excluding patients that might have an increased risk for nonadherence. The revised ProMAS was applied 2 weeks after treatment initiation (baseline) and at 8 months’ follow-up, both in the intervention and in the control group. In the follow-up measurement all patients received the 17-item revised ProMAS questionnaire. The revised ProMAS score at baseline signaled nonadherence risk and showed whether the patient was eligible for an intervention while the control group data were used to assess the predictive values of the revised ProMAS. The effectiveness of the intervention on the revised ProMAS score at follow-up was a secondary outcome measure.

#### Barriers to Adherence

Similar to the nonadherence risk, barriers were assessed at inclusion and follow-up, and in both the intervention group and the control group. The result at baseline informed the infographic and consequently the intervention in those patients in the intervention group that had a revised ProMAS score indicative of a high nonadherence risk. Data at inclusion in the control group were used to assess the predictive values of the barrier questionnaire. The results at follow-up were used to assess the effectiveness of the intervention on the barriers.

#### Adherence

Medication adherence was calculated as the percentage of days covered (PDC) by medication based on pharmacy dispensing data of 1 drug group from therapy initiation to follow-up after 8 months. The denominator of the PDC was the number of days in 8 months from the first dispensing. For the numerator of the PDC we counted the days covered by medication. Gaps in availability due to late follow-up dispensing led to a lower PDC.

We used the PDC in a dichotomized way and as a continuous measure. Applying Haynes’s empirical definition of adequate adherence (ie, at least 80% of drugs taken) to antihypertensive medication, patients with a PDC of at least 80% were labeled as “adherent.” Although it may depend on the specific medication in use, this cut-off point is commonly used in the literature as a critical value for nonadherence [29,30]. As second main outcome, we assessed the effectiveness of the intervention on the PDC as continuous measure.

PDCs were calculated per medication group (Multimedia Appendix 1), except for blood glucose–lowering drugs, which were calculated at the subclass level, for example, biguanides (ATC code A10BA) and sulfonylurea derivatives (ATC code A10BB).

The follow-up period was set at 8 months to include at least two follow-up prescriptions covering 3 months each after the first dispensing for 2 weeks. Within participating pharmacies information on the PDC of included patients was received in an anonymized way for intervention and control pharmacies.

### Outcomes

#### Primary Outcome

The primary outcome was the effectiveness of the intervention on medication adherence (PDC dichotomized as ≥80% of days covered and PDC as a continuous measure) comparing intervention and control group patients after an 8-month follow-up.

#### Secondary Outcomes

- The difference in percentage of patients with an increased risk for nonadherence based on the revised ProMAS between the intervention and control groups after an 8-month follow-up.
- The effectiveness of the intervention on the composite barrier score (See the “Data Analysis” section).
- The positive and negative predictive values of (1) the revised ProMAS score and (2) the barriers profile measured at baseline in the control group in relation to medication adherence at 8 months’ follow-up.

In the study protocol we formulated a secondary outcome relating to medication adherence in the subgroup of patients with a follow-up period of at least one year. As data might be available easily, this would be a way to study whether an effect would sustain. We refrained from this outcome because we found no effectiveness in the primary outcome after 8 months.

### Sample Size

For the sample size calculation we assumed that 60% of the patients at high risk in our sample, based on their revised ProMAS score, would be nonadherent (defined as PDC <80%), with a 20% increase in patients with a PDC ≥80% in the intervention group compared with the control group [31]. Concerning the effect of clustering of patients within pharmacies, we assumed an intracluster correlation of 0.05.

For this trial in the care group setting, we expected at least 14 community pharmacies to participate. The sample size calculation indicated that 39 patients at high risk for nonadherence are needed per pharmacy (power 80%, type 1 error 5%; PASS software version 11).

### Data Analysis

To assess differences between the groups, we performed linear and logistic mixed model multilevel analyses, with adjustment for potential confounders (patient age, gender, diagnosis [diabetes or not]). We planned to control for the number of comedication in chronic use, but lacked the data for this.

We compared differences in medication adherence between the intervention and control groups both as a dichotomous (PDC <80% versus PDC ≥80%) and as a continuous outcome measure. We performed intention-to-treat analyses.

During the study period data from the process evaluation (eg, patient interviews) showed that the intervention was not always applied. Post hoc we performed the same analyses per protocol. From the intervention group we included only those patients from whom we had proof that they actually received an intervention (based on a note about the intervention in the electronic chain system or from questionnaire or interview data from the process evaluation) and compared these with the patients in the control group.

During analysis we discovered much higher adherence rates in the control condition than anticipated. We therefore tested whether the dichotomized adherence differed between all patients starting research medication in control pharmacies and our research sample in the control group that was part of this larger group of patients. Adherence was based on the PDC, assessed in the same way as for the study population. We used a chi-square test.

We compared differences in the revised ProMAS score between the intervention and control groups by a fixed cut-off point (score ≤13 versus >13) and the mean score between the groups at follow-up.

Further secondary analyses assessed the difference in the composite barrier profile score between the intervention and control groups. For this outcome we computed a composite barrier score based on the profile presentation. Each barrier was scored as 1 (indicative for serious barrier), 2 (possible barrier asking for some attention), or 3 (no barrier). We added the 8 barrier scores to form the composite barrier score with a range from 8 to 24, with a higher score indicating fewer barriers.

The predictive values of the revised ProMAS score were computed based on cross tabulation of revised ProMAS and PDC scores dichotomized. All these analyses were performed using SPSS software (version 25; IBM Corp).

Finally, the predictive value of the barrier profile was assessed using machine learning, performed in R [32,33]. The barrier profile consists of 8 individual barrier scores that were used to train a machine learning algorithm to predict adherence at 8 months. The benefit of using a machine learning technique is that combinations of the individual barrier scores that are indicative of nonadherence will be discovered during the training phase of the machine learning model. To create a machine-learned predictive model, the data set was split into training, test, and validation sets. The training set was used for learning the parameters of the predictive model. The test set was used to tune the parameters of a predictive model and the validation set to evaluate the performance of the predictive model. Two-thirds of the sample was used as the test and training sets and one-third for validation.

For evaluating the performance of the predictive model, a 10-fold cross validation was applied [34]. To find the most appropriate machine learning technique for our data, 3 techniques were tested: random forest, kernel support vector machines, and generalized linear models [35,36].

## Results

### Overview

A total of 15 community pharmacies participated (7 pharmacies in the intervention group and 8 in the control group).

In total, pharmacies registered 1405 patients for the study. Of them, 806 completed the first questionnaire and returned a signed informed consent form. Of these 806 patients, pharmacy data were available for 684 patients. We had to exclude 192 patients as they turned out to be no initiators of their drug group according to study criteria (but switchers or restarters) or because they did not start chronic medication from our predefined groups and thus were not eligible for inclusion. So, for analyses we finally had available data from 492 patients. In the intervention group 129/253 patients (51.0%) had a revised ProMAS score, indicating high nonadherence risk; in the control group this concerned 115/239 patients (48.1%). Table 1 describes the sample in terms of the basic patient characteristics; the intervention group and the control group showed no important differences. The questionnaire at 8-month follow-up was filled in by 370 of the 492 patients. Patient inclusion numbers per pharmacy varied from 3 to 107 patients.

**Table 1:** Patient characteristics.

Characteristics	Intervention group (n=253)	Control group (n=239)
Female, n (%)	127 (50.2)	107 (44.8)
Age, mean (SD)	63.7 (10.8)	62.5 (10.6)
On chronic medication before starting the study medication, n (%)	194 (76.7)	185 (77.4)
In a disease management program for diabetes, n (%)	39 (15.4)	43 (18.0)
Revised ProMAS^a^ score, mean (SD)	12.8 (3.3)	12.9 (3.0)
Revised ProMAS score ≤13, n (%)	129 (51.0)	115 (48.1)
Composite barrier score, mean (SD)	19.4 (2.34)	19.3 (2.26)
Second survey completed, n (%)	188 (74.3)	182 (76.2)

^a^ProMAS: Probabilistic Medication Adherence Scale.

### Outcomes

#### Primary Outcome

Our primary outcome, medication adherence after 8 months, was 65.1% (84/129) in the intervention group and 66.1% (76/115) in the control group. There was no significant difference between the intervention group and the control group (B=–0.01; 95% CI –0.59 to 0.57; *P*=.96). Patients with programmed diabetes care showed a significantly better medication adherence (B=1.02; 95% CI 0.21-1.84; *P*=.01).

Analyzing the data considering the PDC as a continuous outcome gave comparable results (effect intervention: B= –0.74; 95% CI –11.0 to 9.5; *P*=.87; effect programmed diabetes care: B=16.3; 95% CI 6.2-26.5; *P*=.002).

In our post hoc per-protocol analysis we compared the medication adherence of 74/129 patients in the intervention group who received an intervention (71 based on records in the information system, and an additional 3 based on data from patient interviews) with the 115 patients in the control group. No significant difference in medication adherence was found for adherence dichotomized (B=0.19; 95% CI –0.50 to 0.89; *P*=.58) and as a continuous outcome (B=4.5; 95% CI –7.8 to 16.9; *P*=.40).

Medication adherence in all patients starting research medication in the control pharmacies during the research period was 47.79% (2471/5170). This differed significantly from the 72.8% (174/239) in our control sample (*P*<0.001).

#### Secondary Outcomes

#### ProMAS Score and Effectiveness of the Intervention

A total of 370 patients filled in the first as well as the second survey. In the intervention group 55.3% (104/188) had a low revised ProMAS score at 8 months’ follow-up, indicative of a high nonadherence risk; in the control group 51.6% (94/182) had a low revised ProMAS score. The average revised ProMAS score was 12.64 and 12.74, respectively (*P*=.77).

Controlling for clustering in pharmacies, the revised ProMAS score at inclusion, age, gender, and disease management program (diabetes or not), the effect of the intervention was nonsignificant (B=0.05; 95 CI –0.46 to 0.57; *P*=.85). Revised ProMAS dichotomized gave comparable results (B=0.16; 95% CI –0.31 to 0.62; *P*=.50).

We assessed the effectiveness of the intervention on the barrier profile. The average score on the barrier profile at follow-up was 19.7 in the control group and 19.9 in the intervention group. Controlling for clustering in pharmacies, the barrier profile at inclusion, age, gender, and disease management program (diabetes or not), the effect of the intervention was nonsignificant (B=0.11; 95% CI –0.49 to 0.71; *P*=.69).

#### Predictive Values of Revised ProMAS

We tested the predictive value of the revised ProMAS and the barrier questionnaire on the data from all 239 patients in the control group (all data are presented in Table 2). In the control group medication adherence was 72.8% (174/239). Of the patients with a low revised ProMAS score predicting a high risk for nonadherence, 33.9% (39/115) had a PDC less than 80% (nonadherent). This was the positive predictive value of revised ProMAS. Conversely, the negative predictive value was 79% (98/124), meaning that 79% of the patients with a high revised ProMAS score not indicative for a high nonadherence risk had a PDC of 80% or more (adherent). The sensitivity of revised ProMAS was 60% (39/65).

**Table 2:** Revised ProMAS score versus medication adherence.

Revised ProMAS^a^ score	Medication adherence		
	Nonadherent	Adherent	Total
≤13	39	76	115
≥14	26	98	124
Total	65	174	239

^a^ProMAS: Probabilistic Medication Adherence Scale.

#### Predictive Values of the Barrier Questionnaire

**Figure 2:**
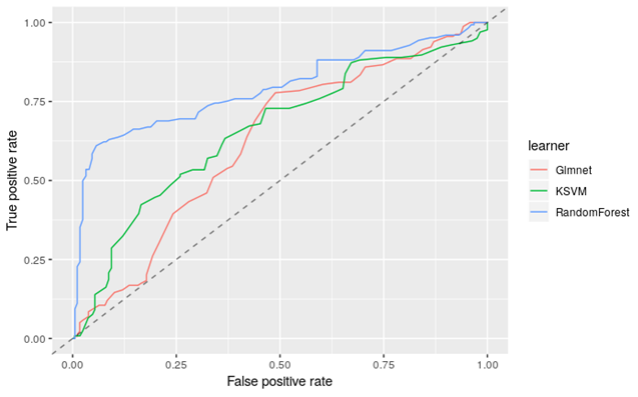
The performance of 3 machine learning techniques (random forest, kernel support vector machines [KSVMs], and generalized linear models). The results are the average outcomes of a 10-fold cross validation and relate to the predictive value of the answers on the barrier assessment survey.

The barrier profile consists of 8 individual barrier scores that were used to train a machine learning algorithm to predict adherence at 8 months. Figure 2 shows the average performance of the 3 applied learners. The best performing learner was the random forest with an area under the curve of 0.795.

Applying the random forest model to the validation set (102/239 patients) yielded the following results: positive predictive value 74%; negative predictive value 80%; and sensitivity 76%.

## Discussion

### Principal Findings

Among initiators of diabetic or cardiovascular drugs, we found no effect of our intervention on medication adherence as measured by pharmacy dispensing data at 8 months’ follow-up. Further, the intervention did not significantly change the secondary outcomes for the medication adherence risk and barriers at 8 months’ follow-up. Instead, receiving programmed diabetic care was associated with higher medication adherence.

The positive and negative predictive values of the risk assessment based on the revised ProMAS questionnaire were 34% and 79%, respectively. Sensitivity was 60%. The predictive values of the barrier questionnaire were 74 and 80%, respectively.

Below we discuss several factors that may have influenced the results: (1) the adherence in the control group was high and left little room for improvement; (2) our study lacked power, as the final patient number eligible for analysis was lower than expected; (3) the fidelity of the pharmacists in the intervention group to perform the intervention was lower than expected (many patients actually did not receive the intervention); (4) the accuracy of the revised ProMAS in predicting nonadherence was limited; (5) the quality of the intervention delivery may have been suboptimal; (6) the opportunities to enhance the impact of the intervention in the care chain were not leveraged.

An important hindrance in our study was—in itself very positive—the finding of the high medication adherence level in the control group: 66.1% (76/115) of the patients at high nonadherence risk had a PDC over 80%. Many patients had an already optimal PDC of 100% from usual care alone, which leaves no room for improvement. Although we have no data proving it, we expect that repeat prescription services and multidose dispensing systems may have led to more patients with a PDC of 100%. When offered more often in study patients, this might be seen as a form of cross over.

Our assumptions for the sample size calculation were based on average adherence data in the literature of 50%. This percentage was confirmed by the adherence of all patients starting research medication in the control pharmacies of 47.79% (2471/5170). The high medication adherence in our sample gives rise to several possible explanations. First, this may be due to selection in the pharmacies during the phase of inviting patients at the first dispensing. Selection bias might also have occurred at the patient level: patients who consider medication adherence important might be more willing to participate in the study. Another explanation of the high adherence rate in our study sample might be the so-called Hawthorn effect: knowing you participate in a study will influence your behavior [37,38]. Finally, the questionnaire used in both the intervention and control groups might have triggered patients to reflect on their behavior, their health, and the importance of medication, resulting in higher adherence rates. Therefore, filling out the questionnaire might be considered an intervention in itself.

We operationalized medication adherence by pharmacy dispense data, which might be criticized. The PDC as a surrogate measure of adherence is a conservative measure for nonpersistence without taking noninitiation into account [9].

Second, the intervention was not always delivered to those patients selected for it. All patients with a revised ProMAS score indicative of a high nonadherence risk had to be offered an intervention with notes in the multidisciplinary electronic file. From the lack of notes and from patient information gathered in the process evaluation, we learned that many eligible patients did not receive an intervention. Clear descriptions, possibilities for flexible time management, simple patient inclusion, and task delegation could increase participation in the intervention [39]. Although the pharmacists agreed to participate, the lack of flexibility relating to the timing of the intervention might have been a barrier to perform it according to the intervention protocol.

In our per-protocol analyses we only included those patients to whom the intervention was actually offered. However, in this smaller sample we did not find an effect.

Third, the number of included patients per month proved to be far lower than anticipated. The research team, therefore, put much effort in supporting the pharmacies to invite patients and we increased the potential of eligible patients by allowing them to be included from GPs other than the participating GP care group. Still, we did not manage to reach the calculated sample size. Moreover, we ended up with a large variation between numbers of included patient per pharmacy. Variation in cluster size requires even higher numbers to achieve the same statistical power level [40]. The final sample size was even further compromised because we had to exclude patients from the analysis who did not fulfil the inclusion criteria. This was mainly due to the stricter criteria for therapy initiation from our study compared with the automated alerts from the pharmacy system.

Fourth, the accuracy of the prediction of nonadherence risk was limited. We preferred to use the revised ProMAS over other adherence questionnaires as it measures behavior and not the beliefs, attitudes, and intentions [22]. The adherence results in our control group showed that 2 out of 3 patients who were offered an intervention would have been adherent at 8 months without an intervention. This is inefficient regarding time and means and dilutes any possible effect from a research perspective. By contrast, with a revised ProMAS sensitivity of 60% in 40% of nonadherent patients we missed the chance to offer these patients an intervention. Although personalized care involves risk stratification, the diagnostic ability of revised ProMAS may not have been sufficient yet. The predictive characteristics of the barrier questionnaire proved to be better.

Fifth, the quality of intervention delivery may have been suboptimal. Before the start of the project, pharmacists followed a 3-hour training in communication skills and intervention delivery. During the intervention period, pharmacists were offered extra training to improve their communication skills for the intervention. Process evaluation showed that this training was mainly used to discuss difficulties in patient inclusion and thus communication skills may not have been developed as expected.

Sixth, collaboration between pharmacy and general practice in reinforcing the intervention did not happen. Moreover, pharmacists did not offer devices to measure blood pressure or blood glucose to the patients, although we offered this as a way to improve patients’ motivation [12]. Interviews during the study period with the pharmacists showed that they experienced a lack of skills to recruit and perform adherence conversation and often lacked time to execute interventions on busy days.

Improvement of our intervention might apply various elements. The basic principle of profiling a patient based on nonadherence risk and barriers for adherence seems to align well with the trend to provide personalized care in general and more specific with the trend to tailor medication adherence interventions to the needs of the patient.

Improving the instrument for patient selection would help to put the energy where it is most beneficial. The positive and negative predictive values of the revised ProMAS were 34% and 79%, respectively. In their review, Lam and Fresco [41] mentioned that the Morisky Medication Adherence Scale has advantages over other self-reporting adherence scales. Tan et al [42] found in their review 2 studies reporting predictive values of the Morisky Medication Adherence Scale. The positive predictive values were 0.41 and 0.71, the negative predictive values were 0.65 and 0.43, respectively [43,44]. Further research could include the evaluation of the individual responses of all patients to the revised ProMAS questionnaire. This would provide more insight into its test characteristics.

Additionally, from dispensing data available in the pharmacy, it should be possible to only provide the intervention to patients on chronic medication not collecting their medication in time.

The assessment of the predictive values of the barrier profile shows better results to target patients than those of the revised ProMAS, indicating that the barriers measured are relevant for medication adherence.

More collaboration in the chain of health care professionals is another possible way to strengthen the intervention. When in the general practice the barrier profile and the data from the pharmacist’s intervention are directly visible in the chain system, the GP and practice nurse can build upon the intervention or at least support and underline it. In our study the intervention was not supported in the general practice.

### Strengths and Limitations

As discussed above, the sample size was an important limitation of our study. We failed to include the patient numbers needed and for about 100 participants medication data could not be linked. Almost 200 patients included were not eligible because they were not a “starter” with one of the trial medications. To increase patient recruitment, we allowed pharmacists who hardly managed to include patients to include patients listed with GPs from another care group. Although we do not expect effect on the outcome, this change in eligibility is a limitation.

Another limitation was that we had to adapt the ProMAS questionnaire for those patients not on medication yet. Consequently, the version we used, excluding questions, was not formally validated as the original version. The measurement of nonadherence risk only shortly after medication initiation may result in a slightly more positive nonadherence risk due to the situation-specific nature of certain items and the decreased chance of the occurrence of these situations in a short period compared with a longer period (eg, When I am away from home, I occasionally do not take my medicine). However, we used a liberal cut-off, thereby lowering the chances of missing any patients at increased risk for nonadherence.

In our research we were not able to control for polypharmacy. Patients on medication and starting with another medication (add-on) are in another situation than patients starting with 1 first medication. While more medication can add more difficulties, it could also enhance adherence to existing regimen(s).

An important strength of our study was the trial design, with pharmacies being randomized and analyses taking patient clustering into account. This was achieved in collaboration with an industrial company providing user-friendly technology and the care professionals with their care group policy makers. This study was carried out in the daily practice after a pilot phase to customize the processes and materials.

Taking less medication by skipping dosages and stopping after a certain period may result in the same PDC indicating nonadherence. So, our PDC data do not allow for conclusion on the type of nonadherence.

### Conclusion

Our tailored intervention for initiators of cardiovascular or diabetes medication did not improve medication adherence compared with usual care. However, interventions tailored to individual barriers of those patients with an increased risk for nonadherence appear to be a good strategy in line with the current policy to personalize care. A study with a better selection of those patients who could benefit and a better implementation of the intervention might well show positive results.

## Appendix

Multimedia Appendix 1 Patients starting medication from the listed ATC codes for cardiovascular or oral blood glucose lowering medication were eligible for inclusion.

Multimedia Appendix 2 CONSORT-eHEALTH checklist (V 1.6.1).

## Abbreviations

ATC: Anatomic Therapeutical Chemical
BOMM: Begeleiding op Maat bij Medicatie
CaZo: Stichting Categorale Zorg voor apothekers in Zuidoost Brabant
CMO: Commissie Mensgebonden Onderzoek
DOH: De Ondernemende Huisarts
GP: general practitioner
PDC: percentage of days covered
ProMAS: Probabilistic Medication Adherence Scale
