# Supplementary material for: Effectiveness of a Pharmacist-Led Web-Based Medication Adherence Tool With Patient-Centered Communication: Results of a Clustered Randomized Controlled Trial
Source: J Med Internet Res. 2022 Apr 7;24(4):e16141. doi: 10.2196/16141 (PMC9030914; doi:10.2196/16141)
Supplement: Multimedia Appendix 1 [file jmir_v24i4e16141_app1.docx]

**Multimedia Appendix 1.** Patients starting medication from the listed ATC codes for cardiovascular or oral blood glucose lowering medication were eligible for inclusion.

- ATC code A10B: oral antihyperglycaemic drugs
- ATC code B01AC: platelet aggregation inhibitors
- ATC code C01A: cardiac glycosides
- ATC code C01D: vasodilators
- ATC code C03: diuretic drugs
- ATC code C07: beta blocking agents
- ATC code C08: calcium channel blockers
- ATC code C09: agents acting on the renin-angiotensin system
- ATC code C10: lipid lowering drugs
